# Adolescent sexual initiation and pregnancy: what more can be learned through further analysis of the demographic and health surveys in the Philippines?

**DOI:** 10.1186/s12889-019-7451-4

**Published:** 2019-08-20

**Authors:** Christine Marie Habito, Cathy Vaughan, Alison Morgan

**Affiliations:** 1 Centre for Health Equity, Melbourne School of Population and Global Health, University of Melbourne, 207 Bouverie Street, Carlton, Victoria 3053 Australia; 2 Gender and Women’s Health Unit, Centre for Health Equity, Melbourne School of Population and Global Health, University of Melbourne, Carlton, Victoria Australia; 3 Maternal, Sexual and Reproductive Health Unit, Nossal Institute for Global Health, Melbourne School of Population and Global Health, University of Melbourne, Melbourne, Victoria Australia

**Keywords:** Adolescent, Teenage, Sexual initiation, Pregnancy, Demographic and health surveys, Sexual and reproductive health

## Abstract

**Background:**

Adolescent pregnancy poses risks to the life of a young mother and her baby, and can affect their health, educational and future employment outcomes. In many low- and middle-income countries like the Philippines, the Demographic and Health Surveys (DHS) Program is among the most reliable and easily accessible sources of demographic and health data for researchers, development workers, and policymakers. Data on adolescent sexual and reproductive health (SRH) are often limited, but in the absence of other sources, there is room to make the most of the adolescent health data gathered by the DHS. The aim of this study is to explore what more can be learned about adolescent sexual initiation and pregnancy through the further analysis of demographic and health data, using DHS data from the Philippines as an example.

**Methods:**

This study conducted trend analysis of DHS data over three survey rounds (2003, 2008 and 2013) to explore the context of adolescent sexual initiation and pregnancy over time. Bivariate and multivariate logistic regression were then used to study associations between adolescent pregnancy experience and selected demographic, socioeconomic and SRH variables using data from the 2013 DHS.

**Results:**

This study found that between 2003 and 2013, proportions of Filipino young women experiencing adolescent sexual initiation and adolescent pregnancy have increased. Multivariate logistic regression affirmed the protective effect of education and belonging to higher wealth quintiles on the risk of adolescent pregnancy. Ever use of contraception was positively associated with adolescent pregnancy but is likely indicative of use after a prior pregnancy, and/or other factors relating to improper/inconsistent contraceptive use.

**Conclusions:**

In the absence of reliable, easily accessible data on adolescent SRH, the DHS data can provide important insights about adolescent reproductive transitions such as sexual initiation and first pregnancy. However, there are limited variables in the datasets that could proxy for other important social determinants which prior studies have linked to adolescent SRH outcomes. There remains a need for timely and targeted collection of quantitative and qualitative data on adolescent SRH that can guide programming and policy intended to foster positive health outcomes during this crucial transition period to adulthood.

**Electronic supplementary material:**

The online version of this article (10.1186/s12889-019-7451-4) contains supplementary material, which is available to authorized users.

## Background

Adolescent pregnancy poses risks to the life of the young mother and her baby, and can compromise their health, educational and future employment outcomes [1]. Sexual initiation in early adolescence increases the risk of adolescent pregnancy [2], especially in settings where unmet need for contraception/family planning and persists. In Southeast Asia, fertility (childbearing) among adolescents – young people between the ages of 10 and 19 [3] and majority of whom are children – has been declining since the 1970s, but recent decades have seen some countries defying this trend. Based on 2017 estimates from the United Nations, adolescent fertility in the Lao People’s Democratic Republic (PDR), the Philippines, Thailand, Cambodia, and Indonesia exceeded the regional average of 44 births per 1000 women 15–19 years old in 2015–2020 [4]. The Philippines, Thailand and Cambodia have seen increasing adolescent fertility beginning between 2000 and 2010, while in Lao PDR and Indonesia, decades of declining adolescent fertility has begun to taper [5].

Current knowledge on factors that specifically influence adolescent sexual and reproductive health (SRH) and particularly unplanned adolescent pregnancy in Southeast Asia is limited. In general, adolescent fertility has been discussed in relation to three key contributors: early marriage, sexual initiation, and contraceptive behaviour [6]. However, early marriage is not as common in Southeast Asia as in sub-Saharan Africa and South Asia. In fact, prior studies have found a trend in the region toward delaying marriage in favour of cohabitation/informal unions [7, 8]. Some studies conducted in Southeast Asian countries report that young people are experiencing sexual initiation in adolescence [9, 10], at younger ages [11–14], and before union formation (i.e. cohabitation or marriage) [12, 15, 16]. There is also evidence that up to one in four marriages in Asia occur after a premarital conception [6], suggesting that many adolescents enter unions earlier than they may have intended to, or been ready for, following a pregnancy [10]. Furthermore, adolescents – especially those who are unmarried and sexually active – often have the greatest unmet need for contraception [17], and face multiple barriers to contraceptive information, access, and use [10, 18], putting them at risk of unplanned pregnancy.

In many countries, few (if any) national level surveys of young people exist. For many low- and middle-income countries, the Demographic and Health Surveys (DHS) Program is among few, if not the only reliable, easily accessible sources of demographic and health data. The DHS is a cross-sectional, nationally representative survey conducted every five years, covering themes such as family planning; maternal, neonatal, and child health and nutrition; HIV/AIDS knowledge, attitudes and behaviour; and women’s empowerment. The sample for a standard DHS includes women (and sometimes men) of reproductive age (typically 15–49 years old), and so each DHS dataset contains valuable data on young people that can be used to inform the work of researchers, development workers, and policymakers. Prior studies have noted that while important and useful, the DHS final reports alone are limited in their ability to guide adolescent reproductive health programmes due to the omission of important indicators, limited reporting of age-disaggregated data, and the exclusion of young adolescents (10–14 years old) and, for some countries, the exclusion of unmarried women from participation in the survey [19, 20]. However, since the DHS Program makes its recoded datasets available, there is an opportunity to learn more from the DHS datasets through undertaking further analysis.

As such, the research question that this study aims to address is: In the absence of other data sources, what more can be learned about adolescent sexual initiation and pregnancy through further analysis of DHS data? Among the Southeast Asian countries that have exhibited increasing adolescent fertility in recent decades, the Philippines presents an interesting case, as it has shown the longest-running, consistent increases in adolescent fertility rates in the region (since the early 2000s) [5]. This study shall, therefore, use DHS datasets from the Philippines as an example. Although not exhaustive, such an exercise can demonstrate how to maximize existing datasets to improve understanding of the contexts within which adolescent sexual initiation and pregnancy are occurring, and help inform adolescent sexual and reproductive health programming and policymaking in other low- and middle-income countries.

## Methods

### The DHS data

The aim of this study is to demonstrate how DHS data can be used to generate added insight on adolescent sexual initiation and pregnancy by examining trend data over several survey rounds, and using quantitative analysis methods not applied in the DHS final reports on data from a single dataset. Data for this study were from the Philippines DHS rounds in 2003, 2008 and 2013 [21–23], which were reviewed and approved by the ICF Institutional Review Board.^1^ Permission to use the data for this study was granted by the DHS Program. Men’s data were only collected in the 2003 Philippines DHS, and so analysis here focuses only on women’s data. In 2003, 13,633 women were interviewed, of whom 2648 were adolescents aged 15–19 years (19.4% of the total sample). In 2008, 13,594 women were interviewed, including 2749 adolescents (20.2% of the total sample), while in 2013, 16,155 women were interviewed, including 3237 adolescents (20.0% of the total sample). More information on the sampling design and survey instruments used in the Philippines DHS can be found in the final reports [24–26].

This study’s main outcome of interest was whether first pregnancy occurred in adolescence, which occurs following sexual initiation (first sex) in adolescence. Using some of the century month code (CMC) dates given by respondents, an estimated age at first pregnancy was derived for each respondent (see Additional file 1). Age at first sex was also reported by respondents as age in completed years, but is prone to error (e.g. social desirability or recall bias) [27]. Where discrepancies emerged between the estimated age at first pregnancy and reported age at first sex, the former was considered more reliable than the latter, and the reported age at first sex was adjusted as conservatively as possible to reflect the logical sequence of these events. Discrepancies were rare [28–31].

### Analysis

This study began by examining the trends in demographic and socioeconomic characteristics of young women aged 15–19 who had experienced adolescent sexual initiation and pregnancy using data from the 2003, 2008 and 2013 Philippines DHS. We defined “adolescent pregnancy” as “conception/pregnancy before age 20”. The second and third parts of the analysis involved descriptive analysis, and bivariate and multivariate logistic regression to study the relationships/associations between the main outcome of interest (experience of adolescent pregnancy) and selected demographic, socioeconomic and SRH variables, while controlling for the effects of all the variables that were included. We made use of Stata Statistical Software (SE) Release 14 for all our analyses of DHS data [32].

Exposure to the risk of adolescent pregnancy begins with sexual initiation in adolescence and ends at age 20. All adolescents are exposed to the risk of sexual initiation, but the risk of adolescent pregnancy is limited to adolescents who have experienced sexual initiation. This risk is greater in contexts where lack of access to family planning information and supplies, and improper use of reliable contraceptive methods are prevalent. It was therefore important to distinguish between the contexts of women who did and did not experience sexual initiation before their 20th birthday. In each DHS, the subpopulation of women aged 15–19 is comprised of respondents who may not have experienced adolescent sexual initiation or pregnancy at the time of their interview, but could still go on to experience these outcomes as adolescents at a later time (i.e. after the survey). Thus, it was logical to conduct analysis that focused on the subpopulation of women who most recently completed adolescence (i.e. women aged 20–24), since all women in this subpopulation had surpassed the age at which the risk of experiencing either outcome ends (i.e. they were no longer exposed to the risk of adolescent sexual initiation and pregnancy). As such, the regression analysis for this study focused on women from the 2013 DHS round who (a) were 20–24 years old, and (b) experienced their first sex during adolescence.

Bivariate and multivariate logistic regression models were used to examine the contexts of young adult women (20–24 years old) who did and did not experience adolescent pregnancy, particularly differences in their demographic and socioeconomic characteristics such as level of education, wealth quintile, and urban/rural residence. Current engagement in paid work was also included in the analysis, as unplanned early pregnancy has been linked to lower daily wages in the Philippines [33]. Aside from these, other variables related to SRH were included in the models, such as timing of first sex relative to first union, age at first menstrual period, and ever use of any method of contraception/family planning, as these are all relevant to adolescent pregnancy outcomes [6, 34, 35]. Pearson’s chi-square test was done between the outcome variable and each independent variable. A variable was retained for possible inclusion in the multivariate analysis if it was significant at the 10% level (*P* <  0.100). Pearson’s R correlation coefficients were then derived to determine if any of the variables were collinear, and any correlated variables were reassessed for relevance and removed from the model as necessary. In the multivariate model, covariates were retained if they were significant at the 5% level (*P* <  0.050) in the bivariate regression or deemed important to the line of inquiry regardless of *P*-value. As a final step, manual backward stepwise regression was done to generate the simplest multivariate model with only the most important variables.

## Results

### What can be learned by comparing different DHS rounds?

More adolescent^2^ women experienced sexual initiation and became pregnant in their teens in 2008 and 2013 relative to 2003, but there were smaller proportions of pregnancies among those with sexual experience (Table 1). There were significant differences in the percentage distribution of adolescents with any sexual experience (*P* <  0.001), and adolescents who had ever been pregnant (*P* = 0.022) between 2003 and 2013. However, focusing only on adolescents with sexual experience, there were also differences in the percentage distribution of adolescent pregnancies in 2008 and 2013 (*P* = 0.038). Further analysis (Table 5 in Appendix 1) verified that the reduction in percentage of adolescent pregnancies between 2003 and 2013 was significant (*P* = 0.014), but that between 2003 and 2008 was not. A closer look at the subset of adolescents below 18 years old only (i.e. those who were still considered children) revealed an upward trend in pregnancy experience, from 3.3% in 2003, to 4.3% in 2008, and 5.0% in 2013, but this was only marginally significant (*P* = 0.051).

**Table 1:** Percentage distribution of adolescents by sexual initiation and pregnancy indicators across three DHS rounds, Philippines, 2003, 2008 and 2013

	Weighted count (Percentage distribution, by column) [95% Confidence Intervals]			
Adolescent sub-population	2003	2008	2013	*P*-value^
Ever had sex				< 0.001
No	2370	2374	2762	
	(89.5%)	(86.4%)	(85.4%)	
	[88.2,90.7]	[84.9,87.7]	[83.9,86.7]	
Yes	278	375	472	
	(10.5%)	(13.6%)***	(14.6%)***	
	[9.3,11.8]	[12.3,15.1]	[13.3,16.1]	
Total	2648	2748	3234	
	(100.0%)	(100.0%)	(100.0%)	
Ever been pregnant, all women				0.022
No	2428	2463	2902	
	(91.7%)	(89.6%)	(89.7%)	
	[90.5,92.8]	[88.3,90.7]	[88.4,90.8]	
Yes	220	286	335	
	(8.3%)	(10.4%)*	(10.3%)*	
	[7.2,9.5]	[9.3,11.7]	[9.2,11.6]	
Total	2648	2749	3237	
	(100.0%)	(100.0%)	(100.0%)	
Ever been pregnant, women with sexual experience only				0.038
No	58	89	138	
	(20.8%)	(23.6%)	(29.1%)	
	[16.5,26.0]	[19.2,28.7]	[25.1,33.5]	
Yes	220	286	335	
	(79.2%)	(76.4%)	(70.9%)*	
	[74.0,83.5]	[71.3,80.8]	[66.5,74.9]	
Total	278	375	472	
	(100.0%)	(100.0%)	(100.0%)	

Note: There are slight discrepancies in weighted count totals due to missing (non-response) data. *P*-values were derived using Pearson’s chi-square test. Asterisks within cells indicate *P*-values derived from further analysis for selected variables which compares each 2008 and 2013 row value to the base year (2003) of that row through bivariate logistic regression, and where *** *P* <  0.001, ** *P* <  0.010, and * *P* <  0.050. Odds ratios and *P*-values from the further analyses are cited in the narratives and shown in table form in Appendix 2

Also compared to 2003, more adolescent women were initiating sex before union in 2008 and 2013. There was a decline over time in both the number and percentage of adolescents with sexual experience who were married, and this was accompanied by a noticeable increase in the proportion of adolescents with sexual experience who had never been married (Table 2-A). Further analysis revealed that never-married adolescents in 2008 were more than two times more likely (in odds ratios, or OR) to have reported sexual experience compared to never-married adolescents in 2003 (OR = 2.119, *P* = 0.002). Also, never-married adolescents in 2013 were more than three times more likely to have reported sexual experience compared to their counterparts in 2003 (OR = 3.692, *P* <  0.001).

**Table 2:** Percentage distribution of adolescents reporting (A) sexual experience and (B) pregnancy experience across three DHS rounds, Philippines, 2003, 2008, and 2013

Subpopulation: adolescents	(A) ADOLESCENT SEXUAL INITIATION Weighted count (Percentage distribution, by column) [95% Confidence Intervals]				(B) ADOLESCENT PREGNANCY Weighted count (Percentage distribution, by column) [95% Confidence Intervals]			
	2003	2008	2013	*P*-value	2003	2008	2013	*P*-value
Marital status				< 0.001				< 0.001
Never-married	31	67	135		16	26	44	
	(11.3%)	(17.8%)**	(28.5%)***		(7.1%)	(9.0%)	(13.3%)**	
	[8.0,15.7]	[13.5,23.0]	[24.3,33.1]		[4.3,11.8]	[5.6,14.2]	[9.9,17.6]	
Married	104	80	68		91	62	63	
	(37.5%)	(21.3%)	(14.4%)		(41.4%)	(21.6%)	(18.8%)	
	[31.2,44.1]	[17.4,25.8]	[11.4,18.0]		[34.4,48.8]	[17.1,27.0]	[14.9,23.5]	
Living with partner	134	204	245		105	177	208	
	(48.3%)	(54.3%)	(51.9%)		(47.6%)	(61.9%)	(62.0%)	
	[41.9,54.7]	[48.7,59.9]	[47.1,56.6]		[40.4,54.9]	[55.7,67.8]	[56.5,67.2]	
Widowed/separated/divorced	8	25	25		8	21	20	
	(3.0%)	(6.6%)	(5.2%)		(3.8%)	(7.4%)	(5.9%)	
	[1.3,7.0]	[4.5,9.7]	[3.4,7.9]		[1.6,8.8]	[4.9,11.1]	[3.6,9.3]	
Level of education				0.002				0.109
Primary or lower	94	88	96		76	72	79	
	(33.9%)	(23.5%)	(20.3%)		(34.4%)	(25.3%)	(23.7%)	
	[27.6,40.9]	[19.5,28.1]	[16.7,24.5]		[27.4,42.3]	[20.6,30.8]	[19.2,28.9]	
Secondary	161	239	314		123	178	217	
	(58.1%)	(63.8%)***	(66.4%)***		(56.1%)	(62.3%)**	(64.9%)**	
	[51.3,64.6]	[58.5,68.9]	[62.0,70.6]		[48.7,63.3]	[56.1,68.1]	[59.6,69.8]	
Higher education	22	47	63		21	35	38	
	(8.0%)	(12.6%)**	(13.3%)**		(9.5%)	(12.4%)	(11.4%)	
	[5.3,11.7]	[9.2,17.1]	[10.5,16.7]		[6.2,14.1]	[8.5,17.8]	[8.5,15.2]	
Wealth quintile				0.015				0.119
Lowest	95	91	108		78	75	80	
	(34.3%)	(24.3%)	(22.8%)		(35.5%)	(26.3%)	(24.0%)	
	[28.0,41.3]	[19.8,29.4]	[18.8,27.3]		[28.4,43.2]	[21.0,32.4]	[19.4,29.3]	
Second	72	87	104		58	71	75	
	(25.8%)	(23.1%)	(21.9%)		(26.2%)	(25.0%)	(22.3%)	
	[20.7,31.6]	[19.0,27.8]	[18.1,26.3]		[20.5,32.7]	[20.2,30.4]	[17.6,27.7]	
Middle	52	79	115		40	63	89	
	(18.8%)	(21.2%)*	(24.4%)***		(18.2%)	(22.1%)*	(26.4%)**	
	[14.4,24.2]	[17.0,26.2]	[20.5,28.7]		[13.3,24.3]	[17.1,28.0]	[21.7,31.7]	
Fourth	38	64	90		30	46	61	
	(13.5%)	(17.0%)*	(19.1%)***		(13.6%)	(16.2%)^+^	(18.2%)*	
	[9.9,18.2]	[12.8,22.2]	[15.3,23.5]		[9.6,18.7]	[11.6,22.1]	[13.8,23.7]	
Highest	21	54	56		15	30	30	
	(7.6%)	(14.4%)	(11.9%)		(6.6%)	(10.4%)	(9.1%)	
	[4.9,11.5]	[11.1,18.6]	[8.9,15.8]		[4.0,11.0]	[7.3,14.8]	[6.0,13.4]	
Total	278	375	472		220	286	335	
	(100.0%)	(100.0%)	(100.0%)		(100.0%)	(100.0%)	(100.0%)	

Source: Appended Philippines DHS 2003, 2008 and 2013 datasets. Note: There are slight discrepancies in weighted count totals due to the application of sampling weights. *P*-values in the columns were derived using Pearson’s chi-square test, and refer to general differences across cells for each variable. Asterisks within cells indicate *P*-values were derived from further analysis for selected variables which compares each 2008 and 2013 row value to the base year (2003) of that row through bivariate logistic regression, and where *** *P* <  0.001, ** *P* <  0.010, * *P* < 0.050, and ^+^ *P* < 0.100. Odds ratios and *P*-values from the further analyses are cited in the narratives

As proportions of never-married adolescent women reporting any sexual experience increased, so too did pregnancy prevalence. A downward trend was observed in the percentage of ever-pregnant adolescents who were married, and this was accompanied by an increase in the percentage of ever-pregnant adolescents who were never-married or cohabiting (Table 2-B). It appears that as the proportion of never-married adolescents with sexual experience increased, so too did the proportion of never-married, ever-pregnant adolescents. Again, further analysis revealed that never-married adolescents in 2013 were significantly more likely than their never-married counterparts in 2003 to have experienced pregnancy (OR = 2.358, *P* = 0.006), although the change between 2003 and 2008 was not significant.

Among adolescent women with sexual experience and some higher education, pregnancy was less likely compared to women with only secondary or primary education (or lower), where changes in sexual experience were accompanied by corresponding changes in pregnancy. Relative to 2003, the percentages of women with some higher education with any sexual experience were higher in 2008 and 2013, and further analysis showed that these year-on-year changes were significant (Table 2-A). However, the changes in 2008 and 2013 in the percentage of adolescents with some college education reporting pregnancy experience were not significant relative to 2003 (Table 2-B). In contrast, relative to 2003, for women with primary education or lower, the changes in sexual experience (Table 2-A) and adolescent pregnancy (Table 2-B) in 2008 and 2013 were not significant, while for women with some secondary education, the increase in sexual experience and adolescent pregnancy were significant.

Adolescent pregnancy affects young women in all wealth quintiles. Percentage distribution of adolescent pregnancies according to wealth quintile mirrored the trends in percentage distribution of adolescents with sexual experience (Table 2-B), where there was in increase in the percentage of adolescent pregnancies coming from the middle and fourth quintiles. In 2003, more than half of all ever-pregnant adolescents belonged to the two lowest wealth quintiles, whereas in 2013, the majority of teenage pregnancies were distributed across the three lowest quintiles. These trends show that as of 2013, adolescent pregnancies in the Philippines are less concentrated in low-income communities than they once were.

Having examined the trends across a decade, the following section presents further analysis of a single DHS dataset, focusing on the subpopulation of women who were 20–24 years old in 2013.

### What can be learned from a single DHS round?

In the 2013 Philippines DHS, there were 2789 female respondents who were 20–24 years old,^3^ of whom 31.0% reported experiencing their first pregnancy in adolescence. Among those who experienced sexual initiation as teenagers (*n* = 1175), the mean age at first sex was 17.6 years, while the mean age at first pregnancy was 17.9 years. Most young adult women who reported experiencing an adolescent pregnancy experienced sexual initiation before their first union, had some secondary education, belonged to the three lowest wealth quintiles, lived in a rural area, and had used some form of contraception in the past. In addition, most of them were not engaged in paid work at the time of interview.

As shown in Table 1, one in ten (10.4%) adolescents who participated in the 2008 DHS had ever been pregnant. In 2013, the subpopulation of women who were 20–24 years old represents women who were 15–19 years old in 2008. Similar analyses have been done using data from specific birth cohorts from different DHS rounds [27]. As such, although not taken from the same sample, there is some indication that relative to the rate captured in the “ever pregnant” indicator (10.4% in 2008), about three times more adolescents from that age group actually became pregnant by the time all of them had turned 20 years old (31.0% in 2013). Furthermore, 73% of all young adult women with any sexual experience (*n* = 1619) had their first sex as teenagers, and almost three-quarters (73.6%; *n* = 1175) of these women also experienced their first pregnancy before their 20th birthday.

### Analysing adolescent sexual initiation and pregnancy

Women who do not have sex before age 20 are not at risk of adolescent pregnancy. As such, the logistic regression analysis focused only on women 20–24 years old who experienced adolescent sexual initiation (*n* = 1175), to better understand the common attributes of those who were exposed to the risk of adolescent pregnancy. Selected demographic and socioeconomic attributes of young adult women aged 20–24 in 2013 (*n* = 2789), broken down into women who (A) experienced adolescent sexual initiation, and (B) experienced adolescent pregnancy, are shown in Table 3.

**Table 3:** Selected demographic and socioeconomic characteristics of women 20–24 years old who experienced adolescent sexual initiation, and adolescent pregnancy, Philippines DHS 2013

Subpopulation: Women 20–24 years old	(A) Adolescent sexual initiation			(B) Adolescent pregnancy			(C) All women
	Percentage (column)	95% C.I.	*P*-value	Percentage (column)	95% C.I.	*P*-value	Wtd. count
Timing of first sex			< 0.001			< 0.001	
First sex at/after first union	12.4%	[10.5,14.5]		14.5%	[12.3,17.0]		1354
First sex before first union	87.6%	[85.5,89.5]		85.5%	[83.0,87.7]		1436
Level of education			< 0.001			< 0.001	
Primary or lower	15.9%	[13.8,18.3]		18.9%	[16.3,22.0]		285
Secondary	57.4%	[54.5,60.3]		61.5%	[58.0,64.8]		1250
Higher	26.7%	[24.1,29.4]		19.6%	[17.0,22.5]		1255
Wealth			< 0.001			< 0.001	
Lowest	20.3%	[17.8,23.2]		24.2%	[21.1,27.6]		382
Second	23.5%	[20.9,26.2]		24.7%	[21.7,27.9]		485
Middle	21.7%	[19.2,24.4]		21.9%	[19.0,25.0]		590
Fourth	21.2%	[18.6,24.1]		19.4%	[16.5,22.6]		661
Highest	13.3%	[11.2,15.7]		9.9%	[7.9,12.4]		671
Residence			0.001			0.009	
Urban	51.9%	[48.8,54.9]		49.5%	[45.8,53.1]		1558
Rural	48.1%	[45.1,51.2]		50.5%	[46.9,54.2]		1231
Region			0.001			0.550	
NCR	16.4%	[13.9,19.2]		16.4%	[13.6,19.7]		567
CAR	1.7%	[1.3,2.3]		1.7%	[1.2,2.4]		48
Ilocos Region	4.2%	[3.2,5.5]		4.6%	[3.4,6.2]		110
Cagayan Valley	5.0%	[4.0,6.2]		5.2%	[4.0,6.8]		117
Central Luzon	9.9%	[8.0,12.1]		9.6%	[7.3,12.6]		263
CALABRAZON	12.0%	[9.9,14.4]		11.3%	[9.1,14.1]		376
MIMAROPA	2.2%	[1.6,3.0]		2.3%	[1.6,3.4]		54
Bicol	5.0%	[3.9,6.3]		4.4%	[3.3,5.8]		127
Western Visayas	5.9%	[4.7,7.5]		5.8%	[4.4,7.6]		159
Central Visayas	6.5%	[5.2,8.1]		5.8%	[4.4,7.7]		180
Eastern Visayas	3.5%	[2.6,4.8]		3.5%	[2.4,5.0]		77
Zamboanga Peninsula	4.7%	[3.8,5.9]		5.1%	[3.9,6.6]		133
Northern Mindanao	4.6%	[3.3,6.3]		4.5%	[3.1,6.5]		131
Davao	6.3%	[5.1,7.9]		6.7%	[5.0,8.8]		153
SOCCSKSARGEN	5.1%	[4.0,6.5]		5.5%	[4.0,7.4]		126
Caraga	4.3%	[3.7,5.0]		4.2%	[3.4,5.1]		88
ARMM	2.7%	[2.0,3.7]		3.3%	[2.4,4.6]		80
TOTAL	1175 (100.0%)			865 (100.0%)			2789
Percentage (row)	72.6%			31.0%			100.0%

Note: *P*-values were derived using Pearson’s chi-square test

#### Adolescent sexual initiation

Bivariate analysis (not shown) revealed that most basic demographic and socioeconomic attributes of women 20–24 years old who experienced adolescent sexual initiation were statistically different from those who did not experience adolescent sexual initiation. Multivariate logistic regression showed that adolescent sexual initiation was less likely with each higher level of education, and wealth quintile, although the difference in odds between women from the lowest and the second wealth quintiles was not significant. Adolescent sexual initiation was also less likely among women who lived in rural areas, but this was only marginally significant (*P* = 0.059) (Table 6 in Appendix 2).

#### Adolescent pregnancy

Looking first at unadjusted odds ratios (Table 4-A), adolescent pregnancy was significantly more likely among women who had ever used any method of contraception, whether it was more than five years ago (*P* <  0.001) or within the last five years (*P* <  0.001). Adolescent pregnancy was also more likely among women who lived in rural areas compared to urban areas (*P* = 0.010). Conversely, adolescent pregnancy was less likely among women who belonged to higher wealth quintiles compared to the lowest; had higher levels of education relative to those with only primary education or lower; experienced adolescent sexual initiation before their first union (*P* = 0.001); and were engaged in paid work at time of interview (*P* = 0.006).

**Table 4:** Multivariate analysis results for adolescent pregnancy experience and selected demographic and socioeconomic characteristics, women 20–24 years old, Philippines DHS 2013

Outcome: Experienced adolescent pregnancy	(A) Unadjusted odds ratio, full (95% C.I.)	*P*-value	(B) Adjusted odds ratio, full (95% C.I.)	*P*-value	(C) Adjusted odds ratio, reduced (95% C.I.)	*P*-value
Sexual initiation before first union (base: Sexual initiation at or after first union)	0.408 (0.240, 0.692)	0.001	0.611 (0.347, 1.076)	0.088	0.616 (0.352, 1.080)	0.091
Level of education (base: Primary or lower)						
Secondary	0.521 (0.324, 0.838)	0.007	0.627 (0.375, 1.047)	0.075	0.608 (0.363, 1.019)	0.059
Higher	0.165 (0.101, 0.271)	< 0.001	0.223 (0.125, 0.396)	< 0.001	0.219 (0.123, 0.390)	< 0.001
Wealth quintile (base: Lowest)						
Second	0.486 (0.304, 0.775)	0.002	0.593 (0.361, 0.973)	0.039	0.576 (0.352, 0.944)	0.029
Middle	0.408 (0.255, 0.653)	< 0.001	0.549 (0.326, 0.922)	0.023	0.534 (0.320, 0.891)	0.016
Fourth	0.291 (0.182, 0.466)	< 0.001	0.517 (0.294, 0.909)	0.022	0.497 (0.292, 0.848)	0.010
Highest	0.172 (0.103, 0.288)	< 0.001	0.365 (0.193, 0.689)	0.002	0.355 (0.195, 0.644)	0.001
Rural residence (base: Urban residence)	1.445 (1.094, 1.909)	0.010	1.038 (0.752, 1.433)	0.821	–	–
Age at first menstrual period (base: 10 years old or below)						
11 to 12 years old	0.601 (0.251, 1.438)	0.252	0.436 (0.169, 1.123)	0.085	–	–
13 to 14 years old	0.888 (0.374, 2.110)	0.788	0.562 (0.219, 1.441)	0.230	–	–
15 years old and above	0.793 (0.335, 1.881)	0.599	0.463 (0.180, 1.189)	0.109	–	–
Ever used any method of contraception/family planning (base: Never used any method)						
Last used more than five years ago	2.491 (1.641, 3.780)	< 0.001	2.784 (1.802, 4.302)	< 0.001	2.829 (1.835, 4.360)	< 0.001
Last used within the last five years	2.570 (1.859, 3.553)	< 0.001	2.825 (2.001, 3.987)	< 0.001	2.855 (2.022, 4.030)	< 0.001
Currently engaged in paid employment (base: Not currently engaged in paid work)	0.685 (0.524, 0.895)	0.006	0.919 (0.680, 1.240)	0.579	–	–
Sub-population size (n), weighted	1175					

Adjusted odds ratios were then derived to factor in the effect of all covariates on the likelihood of experiencing adolescent pregnancy (Table 4-B). Ever using contraception remained positively related to adolescent pregnancy, with dolescent pregnancy almost three times more likely among women who had used some form of contraception in the past, irrespective of whether this was within the last five years or more than five years ago (*P* <  0.001 for both). The protective effect of higher levels of education on the likelihood of experiencing adolescent pregnancy was still evident, although the odds and significance were slightly tempered after adjusting for other covariates. Compared to those who had only some primary education or lower, women with some higher education were 78% less likely to have reported experiencing adolescent pregnancy (OR = 0.223, *P* <  0.001), while those with some secondary education were 37% less likely to have experienced adolescent pregnancy, although this was only marginally significant (OR = 0.627, *P* = 0.075). There remained a negative association between adolescent pregnancy experience and each higher wealth quintile, although the odds and significance were also tempered after adjusting for the effects of other covariates.

In the third, reduced model (Table 4-C) adolescent pregnancy remained almost three times more likely among women who had ever used any method of contraception, regardless of the timing of use (*P* <  0.001 for both). The likelihood of adolescent pregnancy was still significantly lower with each higher level of education, and wealth quintile, although the association between adolescent pregnancy and having some secondary education remained only marginally significant (*P* = 0.059).

## Discussion

This study sought to explore what could be learned about adolescent sexual initiation and pregnancy through DHS data. Using three DHS datasets from the Philippines as an example, we explored what can be ascertained about adolescent sexual initiation and pregnancy in the absence of specialised surveys of adolescent SRH. We investigated trends in adolescent sexual initiation and pregnancy across DHS rounds, and associations between adolescent pregnancy and known determinants using a single DHS dataset. Using DHS data from the Philippines is timely, as the implementation of the Responsible Parenthood and Reproductive Health Act of 2012 (RH Law) just commenced in late 2017. Studies such as this can help to establish a baseline of the SRH situation in the country for the continuous development of programmes and policy.

### Discussion of findings

Descriptive analysis of trend data from adolescent women aged 15–19 during the 2003, 2008, and 2013 DHS rounds showed there were more adolescents initiating sex as teens, and before their first union in 2008 and 2013 compared to 2003. This is despite results from another survey suggesting four out of five Filipino youth still believe it is “important/very important” for a woman to remain a virgin until marriage [11]. Such findings highlight the disconnect between prevailing conservative social norms surrounding sex, and the real lives of Filipino adolescents. Indeed, studies from the Philippines have found there remains social pressure for young Filipino women to project an image of chastity, feigning ignorance about sex and contraception – including not discussing contraception with their partner [36] or parents [37]. Yet, qualitative researchers have also noted a shift in young people’s relationships compared to previous generations, where formal courtship and introduction to parents are forgone in favour of “informal and casual encounters” (p.63) with friends [36], and where alcohol is often cited as catalysing unplanned and unprotected sex [36, 38]. It is important to acknowledge that Filipino adolescents are having sex both within and outside of marriage and informal unions, especially since the legal age of consent to sex is 12 [39]. It is imperative that comprehensive sexuality education begins before the onset of puberty, and that SRH policy and legislation supports the provision of SRH information and services throughout adolescence to ensure informed decision-making when young people experience sexual initiation.

The trend data also suggested more women with sexual experience have been able to avoid or delay pregnancy in 2008 and 2013 compared to 2003. However, this does not diminish the fact that more than two out of three adolescents with any sexual experience reported experiencing adolescent pregnancy in 2013. Given the range of contraceptive methods publicly available from different sources, it is possible for adolescent sexual experience and pregnancy to not necessarily be congruent outcomes, but they often are here. In 2013, almost all Philippines DHS respondents knew at least one method of modern contraception [26], but this awareness did not seem to translate to use, especially among teenage and young adult women. A study using DHS data found unmet need for contraception in the Philippines was highest among women 15–19 years old, and those who were sexually-active and never-married [17], noting that barriers to contraceptive access and use could be better understood through further qualitative inquiry. The RH law was intended to strengthen access to SRH information and services/supplies for all, “regardless of age, sex, disability, marital status, or background” [40], but adolescents below 18 years old are required to provide written consent from a parent or guardian [40].^4^ Within this context, our findings highlight the need for policies that remove known and potential barriers to Filipino adolescents’ access to SRH services and supplies.

DHS data from a single round showed that especially for never-married adolescent women, an increase in the proportion of respondents reporting any sexual experience was accompanied by an increase in adolescent pregnancy, but this relationship was less linear among adolescents with some college education. This was in line with the findings from the trend analysis, which indicated weaker links between adolescent sexual initiation and pregnancy among those with some college education. These findings suggest that level of education is still an important variable in understanding adolescent pregnancy outcomes. Further analysis through logistic regression reaffirmed findings from other studies regarding the protective effect of higher levels of education on adolescent pregnancy outcomes [1, 6], and indicated that staying in school for longer may be disrupting (or at least delaying) the onset of childbearing, although DHS data are limited in their ability to expound further on this. Another explanation is that adolescent pregnancy may also result in school dropout. This would make young women who *do not* get pregnant in their teens more likely to continue their education, and could also explain this result. If basic education policies are to become more responsive to the evolving contexts and SRH needs of adolescents, the relationship between education and adolescent SRH outcomes needs to be better understood and clarified in the country context. Especially with the implementation of age-appropriate comprehensive sexuality education in schools – which is mandated by the RH law – it would be good for indicators pertaining to adolescent SRH outcomes to be assessed and measured to track progress and ensure accountability of the schools toward achieving better SRH outcomes for their students.

Wealth was also inversely related to the likelihood of adolescent pregnancy. Although the results from the descriptive analysis of trend data showed that adolescent pregnancy was less concentrated in lower wealth quintiles in 2013 relative to 2003, the reduced logistic regression model revealed that there were markedly lower odds of experiencing pregnancy for women from the highest wealth quintile compared to those in the lowest. This shows that though adolescent pregnancy was occurring in all wealth quintiles, it was still more likely to happen to young women from poorer families. These findings reinforce the contribution of wealth to achievement of positive health outcomes, and conversely, its role in perpetuating negative ones for those who are already disadvantaged. For young people living in poverty, with less educational and earning opportunities, becoming a parent in adolescence further undermines their potential to improve their economic status and quality of life [41]. However, a prior study done in the Philippines (also using DHS data) cautions that “status variables” such as education and wealth may not be complete proxies for women’s autonomy or empowerment [42]. These findings suggest there is merit in looking deeper into the lived experiences of adolescents, as there may be elements of young women’s autonomy not captured within survey variables such as these. They also highlight the need for targeted public spending to narrow the gap between people from the lowest and higher wealth quintiles when it comes to access to essential SRH services and information.

Finally, the results indicated a significantly higher likelihood of adolescent pregnancy among women who had ever used any method of contraception – an outcome which appears to be counterintuitive. However, an explanation for this is that the variable does not capture the timing and consistency of contraceptive use. As the survey design is cross-sectional and not longitudinal, these results are indicative of association and not causation, and we cannot determine whether pregnancy or use of contraceptives occurred first. Therefore, it is possible the higher odds of adolescent pregnancy reflected in these models are due to contraceptive use *after* an adolescent pregnancy (to prevent or delay a repeat pregnancy) [42], reliance on less effective methods, improper or inconsistent use, social desirability bias, or any combination of these. This underscores the importance of investigating further adolescents’ contraceptive use behaviours (whether married or unmarried), and improving understanding of the context of adolescents’ relationships and daily lives so that SRH policies are evidence-based, and SRH programme interventions are provided to adolescents in the most enabling environment possible.

### Strengths and limitations

This study was able to present descriptive analysis of Philippines DHS data on adolescent sexual initiation and adolescent pregnancy in reference to demographic and socioeconomic variables over three DHS rounds within the span of a decade (2003–2013), which is only done in some DHS final reports for selected variables. Furthermore, in the regression analyses, this study focused on first *pregnancy* as opposed to first *birth* as the outcome variable, which allowed the inclusion in the analysis of women who had been pregnant before but whose first pregnancy (and for some, all pregnancies) had not resulted in livebirth (pregnancy termination). This also made it possible to validate the consistency of the reported age at first sex with reported age at first birth. Since the analysis for this study was completed, data from the 2017 Philippines DHS has been made publicly available, indicating a decline in the percentage of adolescents 15–19 years old who have begun childbearing, from 10.1% in 2013 (confidence interval, CI = [9.0,11.4]), to 8.6% in 2017 (CI = [7.4,9.8]; *P* = 0.066) [26, 43]. Further analysis of the 2017 dataset such as that conducted here could be useful for understanding what factors might have contributed to this reduction, so that efforts to continue to address unplanned adolescent pregnancy can be guided accordingly.

We acknowledge significant limitations, including lack of data on young men, difficulty in interpreting specific associations with variables such as contraceptive use, and in the biases inherent in the DHS. As adolescent pregnancy is any pregnancy before age 20, in the logistic regression, we used retrospective data from women who were 20–24 years old. However, we note that prior studies recommend caution in interpreting adolescent reproductive transitions based on retrospective survey data, due to possible recall and social desirability bias, and possible errors in imputation [27]. Aside from imputation done by the DHS, this analysis also applied the authors’ own imputations based on CMC dates given by respondents (see Additional file 1) to adjust inconsistencies in events; in these few instances, some errors are possible in the derivation of estimated ages at first sex and pregnancy.

## Conclusion

In the absence of reliable, easily accessible data on adolescent SRH, further analysis of DHS data can provide additional insights about trends in adolescent sexual initiation and pregnancy, and identify demographic and socioeconomic attributes that might be associated with these outcomes. This analysis was able to frame trends in adolescent pregnancy in reference to subpopulations of women with sexual experience, which is an important demographic to consider in addressing unmet need for contraception among adolescents. In addition, analysis of data from a single DHS round through multivariate logistic regression reaffirmed the protective effect of education on the risk of adolescent pregnancy, and highlighted the need to strengthen SRH services for young people from resource-poor backgrounds, as they were at greater risk of experiencing unplanned pregnancy. The findings from these analyses could be useful for guiding policy in the Philippines, particularly for SRH service delivery and basic education programming.

However, there were limited variables in the DHS datasets that could proxy for other important determinants which prior studies have linked to adolescent SRH outcomes, and adolescent pregnancy specifically. The variables that were available could only establish association, and so had limited ability to explain why certain relationships exist. While further analysis of DHS data does provide useful information, there are some important aspects of adolescent sexual initiation and pregnancy that are not captured or elaborated in the surveys, such as wantedness and context of first sex, contraceptive use during first sex, pregnancy intention for first pregnancy, and autonomy/decision-making in relationships for unmarried, sexually active adolescents. A deeper understanding of adolescents’ relationships and their progression through reproductive transitions are crucial to ensuring that SRH programmes and policy are relevant to their contexts and lived realities. There remains a need for timely and targeted collection of quantitative and qualitative data on adolescent SRH that can guide programming and policy intended to foster positive health outcomes during this crucial transition period to adulthood.

### Additional file

Additional file 1: Authors’ derivation of estimated ages at first sex and first pregnancy using DHS data from the Philippines in Stata. (DOCX 42 kb)

## Data Availability

The data used in this study are available to download for free from the DHS Program website, following registration for and approval to access. Data can be accessed at the following URLs following approval from the DHS Program (which can also be done through these links): https://dhsprogram.com/data/dataset/Philippines_Standard-DHS_2003.cfm?flag=0 (2003 Philippines DHS); https://dhsprogram.com/data/dataset/Philippines_Standard-DHS_2008.cfm?flag=0 (2008 Philippines DHS); and https://dhsprogram.com/data/dataset/Philippines_Standard-DHS_2013.cfm?flag=0 (2013 Philippines DHS).

## Appendix 1

*Further analysis of* Table 1.

**Table 5:** Bivariate logistic regression results, adolescent sexual initiation and pregnancy by survey year, 2003, 2008 and 2013

	Unadjusted odds ratio	95% C.I.	*P*-value
Ever had sex			
Survey year (base: 2003)			
2008	1.346	(1.125, 1.610)	0.001
2013	1.459	(1.226, 1.738)	< 0.001
Ever been pregnant			
Survey year (base: 2003)			
2008	1.283	(1.053, 1.561)	0.013
2013	1.275	(1.049, 1.549)	0.015
Ever been pregnant, sexually experienced			
Survey year (base: 2003)			
2008	0.851	(0.576, 1.257)	0.418
2013	0.642	(0.450, 0.915)	0.014

## Appendix 2

*Further analysis of adolescent sexual initiation*

**Table 6:** Multivariate logistic regression results, adolescent sexual initiation and selected characteristics, women 20–24 years old, 2013

Outcome: Experienced adolescent sexual initiation	(A) Unadjusted odds ratio, full (95% C.I.)	*P*-value	(B) Adjusted odds ratio, full (95% C.I.)	*P*-value
Level of education (base: Primary or lower)				
Secondary	0.613 (0.465, 0.808)	0.001	0.724 (0.543, 0.967)	0.029
Higher	0.174 (0.131, 0.231)	< 0.001	0.258 (0.189, 0.354)	< 0.001
Wealth quintile (base: Lowest)				
Second	0.788 (0.604, 1.028)	0.078	0.977 (0.740, 1.289)	0.868
Middle	0.455 (0.348, 0.595)	< 0.001	0.604 (0.453, 0.806)	0.001
Fourth	0.362 (0.279, 0.471)	< 0.001	0.594 (0.437, 0.806)	0.001
Highest	0.182 (0.138, 0.240)	< 0.001	0.314 (0.227, 0.436)	< 0.001
Rural residence (base: Urban residence)	1.323 (1.118, 1.566)	0.001	0.838 (0.697, 1.007)	0.059
Sub-population size (n), weighted	**2789**			

## Abbreviations

DHS: Demographic and Health Surveys
RH Law: Responsible Parenthood and Reproductive Health Law of 2012
SRH: sexual and reproductive health

## Footnotes

1 “Protecting the Privacy of DHS Survey Respondents”, available at https://www.dhsprogram.com/What-We-Do/Protecting-the-Privacy-of-DHS-Survey-Respondents.cfm

2 The terms “adolescent(s)”, “teenage(r)” and “teens” are used interchangeably from here on, and refer to young women who are 15–19 years old.

3 Referred to interchangeably as “young adults” from here on.

4 Rule 4 – Service Delivery Standards, Chapter 2 – Provision and Financing of Care, Revised Implementing Rules and Regulations (IRR) of the Responsible Parenthood and Reproductive Health Act of 2012 (R.A. No. 10354).
