# Supplementary material for: Adolescent sexual initiation and pregnancy: what more can be learned through further analysis of the demographic and health surveys in the Philippines?
Source: BMC Public Health. 2019 Aug 20;19:1142. doi: 10.1186/s12889-019-7451-4 (PMC6701073; doi:10.1186/s12889-019-7451-4)
Supplement: Supplementary file 1 — Authors’ derivation of estimated ages at first sex and first pregnancy using DHS data from the Philippines in Stata. (DOCX 42 kb) [file 12889_2019_7451_MOESM1_ESM.docx]

# Supplementary material 1

The following describes the process of determining the estimated age at first sex, and estimated age at first pregnancy using DHS data from the Philippines.

## Deriving estimated age at first pregnancy

Ever-pregnant respondents (everpreg = 1) reported how many months each pregnancy was carried before livebirth or termination (nmp_fp), and for women who were pregnant at interview, number of months pregnant at the time of interview (nmp_cp).

We sought to derive an estimated age at first pregnancy (eafp) by determining how old each respondent was at the time they conceived/got pregnant for the first time. In deriving this variable from the available DHS data, there were several groups of women to consider:

First, there were women who had **ever had a livebirth** (egb = 1). The estimated age at first pregnancy was derived by subtracting the number of months pregnant for the first pregnancy (nmp_fp) from the century month code (CMC) date of birth of the first child born alive (dob_fc), subtracting the CMC date of birth of the respondent (dob) from the difference, and dividing the result by 12 to get the exact age of the respondent (in years, with decimal) at the time that the first child was conceived (Formula 1).

gen eafp_gb = .

1. replace eafp_gb = ((dob_fc - nmp_fp) - dob)/12 if egb == 1 & nmp_fp < 99

Where:

eafp_gb = Estimated age at first pregnancy for women who have given birth at least once before

dob_fc = Date of birth of first child (in CMC)

nmp_fp = Number of months pregnant for first pregnancy

dob = Date of birth of respondent (mother)

egb = Dummy variable for women who have ever given birth

Second, there were women who **had not had a livebirth before (egb = 0) but were pregnant for the first time ever at the time of interview** (cpreg_fc = 1). The estimated age at first pregnancy was derived by subtracting the reported number of months pregnant at the time of interview (nmp_cp) from the CMC date of the interview (doint), subtracting the respondent’s CMC date of birth (dob) from the difference, and dividing the result by 12 to get the exact age of the respondent (in years, with decimal) at the time that the first child was conceived (Formula 2).

gen eafp_cp = .

1. replace eafp_cp = ((doint - nmp_cp) - dob)/12 if cpreg_fc == 1

Where:

eafp_cp = Estimated age at first pregnancy for women who are currently pregnant

doint = Date of interview (in CMC)

nmp_fp = Number of months pregnant for first pregnancy

dob = Date of birth of respondent (mother)

cpreg_fc = Dummy variable for women who are currently pregnant with their first child

Third, there were women who **had not had a livebirth before (egb = 0), but whose first pregnancy was terminated** (which includes miscarriages, stillbirths, and abortions). There are two further sub-groups within this group: women who were pregnant at the time of interview, and women who were not pregnant at the time of interview. For all terminated pregnancies, respondents reported the CMC date of pregnancy termination. In this case, we were interested in the CMC date of pregnancy termination for women whose first pregnancy was terminated (dopt1). Respondents also provided the number of months the pregnancy lasted before termination (nmp_fp). The estimated age at first pregnancy among women whose first pregnancy was terminated (eafp_pt) was derived by subtracting the number of months pregnant (nmp_fp) from the CMC date of pregnancy termination of the first pregnancy (dopt1), subtracting the respondent’s CMC date of birth from the difference (dob), and dividing the result by 12 to get the exact age of the respondent (in years, with decimal) at the time that the first child was conceived (Formula 3).

gen eafp_pt = .

1. replace eafp_pt = ((dopt1 - nmp_fp) - dob)/12 if fpt == 1 & nmp_fp < 99

Where:

eafp_pt = Estimated age at first pregnancy for women who have ever had a terminated pregnancy

dopt1 = Date of first pregnancy termination (in CMC)

nmp_fp = Number of months pregnant for first pregnancy

dob = Date of birth of respondent (mother)

fpt = Dummy variable for women whose first pregnancy ended in termination

The lowest value resulting from eafp_gb, eafp_cp, or eafp_pt (i.e. the age of the respondent when the first of these events occurred, either a livebirth, current pregnancy, or pregnancy termination) is then designated as the estimated age at first pregnancy (eafp) using the following code:

gen eafp = .

replace eafp = eafp_gb if egb == 1

replace eafp = eafp_cp if cpreg_fc == 1

replace eafp = eafp_pt if (egb == 0 & eafp_pt != .) ///

| (eafp_pt < eafp_gb & eafp_gb != .) ///

| (eafp_pt < eafp_cp & eafp_cp != .)

Where:

eafp = Estimated age at first pregnancy

eafp_gb = Estimated age at first pregnancy for women who have given birth at least once before

egb = Dummy variable for women who have ever given birth

eafp_cp = Estimated age at first pregnancy for women who are currently pregnant

cpreg_fc = Dummy variable for women who are currently pregnant with their first child

eafp_pt = Estimated age at first pregnancy for women who have ever had a terminated pregnancy

## Deriving estimated age at first sex

The DHS Program has standardized editing and imputation approaches for inconsistencies/discrepancies in their datasets [1]. Of particular relevance to this analysis are the inconsistencies between reported age at first sex, and the DHS’s imputed age at first birth (v212, fb). Respondents had a reported age at first sex (v525) and **imputed** (by the DHS) age at first sex (v531, fs, fsex), which was given as age in completed years – a whole number value corresponding to the respondent’s age (in years) at first sex, and one that is likely prone to error/bias [2].^[[1]](#footnote-2)^ For this reason, this analysis attempts to derive a more reliable, **derived** age at first sex (afs), which is inferred based on age at first birth and estimated age at first pregnancy data – events that can be considered more salient than first sex, and were imputed based on CMC date of first birth. First, we take note of the DHS’s “rule of one”:

“The editing rules allow responses to be changed to another valid response only in situations where it is clear that the new response is correct. In other situations, the inconsistent item of data is changed to the 'inconsistent' code. One special rule, known as the 'rule of one', is used for some questions. This rule allows a response to be changed by one unit (one month or one year depending on the unit of response to the question) if the modified response will be consistent with the other related data.” (Croft 1991, page 6)

In some cases, the reported age at first sex is inconsistent with this study’s estimated age at first pregnancy (eafp, computed above), which is not a realistic order of events. Indeed, in the sample, there were 1,032 respondents whose reported age at first sex (v525) and imputed age at first sex (v531) were greater than their estimated age at first pregnancy (eafp). However, in all these cases, the discrepancy between reported and imputed age at first sex, and estimated age at first pregnancy were less than one year (i.e. the greatest discrepancy was equivalent to 8 months). To address this, this analysis made use of the DHS’s “rule of one,” and applied it to the imputed age at first sex, but only to observations that had not had the rule applied to it before (i.e. v532 != 3, where v532 is the flag for inconsistent responses to v531) (Formula 4). This entailed subtracting one unit (in this case, one year) from the reported/imputed age at first sex to come up with the derived age at first sex, which was inferred based on other important reproductive events. Meanwhile, for observations where the rule of one had already applied by the DHS (i.e. v532 = 3) to reported (v525) and imputed (v531) age at first sex, it was determined that the most conservative additional adjustment that could be made to age at first sex for these cases was to assume that first sex occurred – at the latest – one month prior to the first pregnancy (eafp). The smallest unit of CMC dates are months, and one month is equivalent to 0.08333, or one-twelfth of a year (Formula 5).

gen afs = gen afs = .

replace afs = fsex if fsex != .

1. replace afs = fsex - 1 if (eafp < fsex & fsex != .) & v532 != 3
2. replace afs = eafp - 0.08333 if (eafp < fsex & fsex != .) & v532 == 3

From the derived age at first sex (afs), an estimated age at first sex (eafsex) is then generated. By default, estimated age at first sex (eafsex) was set to be equal to the derived age at first sex (afs) (Formula 6).

gen eafsex = .

1. replace eafsex = afs if afs != .

1. See v525 for the original reported values, v531 for the imputed values [↑](#footnote-ref-2)
